# TTF-1 Positive Primary Small Cell Carcinoma of the Breast: A Case Report and Review of the Literature

**DOI:** 10.3389/fendo.2020.00228

**Published:** 2020-04-29

**Authors:** Hinda Boutrid, Mahmoud Kassem, Gary Tozbikian, Evan Morgan, Julia White, Manisha Shah, Jeffrey Vandeusen, Sagar Sardesai, Nicole Williams, Daniel G. Stover, Maryam Lustberg, Robert Wesolowski, Vinay Pudavalli, Terence M. Williams, Bhavana Konda, Stephanie Fortier, David Carbone, Bhuvaneswari Ramaswamy, Mathew A. Cherian

**Affiliations:** ^1^Stefanie Spielman Comprehensive Breast Cancer, The Ohio State University, Columbus, OH, United States; ^2^Division of Medical Oncology, Comprehensive Cancer Center, The Ohio State University Medical Center, Columbus, OH, United States; ^3^Department of Pathology, The Ohio State University, Columbus, OH, United States; ^4^Department of Radiation Oncology, Comprehensive Cancer Center, The Ohio State University Medical Center, Columbus, OH, United States; ^5^Department of Neurological Surgery, Comprehensive Cancer Center, The Ohio State University Medical Center, Columbus, OH, United States

**Keywords:** TTF-1, small cell cancer, breast cancer, PDL-1, neuroendocrine cancer

## Abstract

Primary small cell carcinoma of the breast (SCCB) is a rare tumor subtype comprising <0.1% of all breast carcinomas. Here we present a case of thyroid transcription factor-1 (TTF-1) positive SCCB that recurred within 3 years of diagnosis in the lung and lymph nodes. Given the small number of cases, no clear guidelines exist on the appropriate management of patients with these aggressive tumors. We present a case study and review the current literature to highlight the knowledge gaps and needs of patients with these rare tumors. A 50-year-old premenopausal woman with no family history, presented with a palpable right breast mass. Biopsy was consistent with primary SCCB that was poorly differentiated, positive for synaptophysin and chromogranin and TTF-1 and presence of ductal carcinoma *in situ* component showing neuroendocrine differentiation. Imaging with PET, CT, and MRI brain excluded any other sites of primary disease. She underwent a right lumpectomy with axillary lymph node dissection and was treated with adjuvant cisplatin-based chemotherapy and concurrent radiation therapy. Thirty-four months later, routine scans showed a new right lower-lobe lung nodule and an enlarged sub-carinal node that was proven to be poorly differentiated neuroendocrine cancer. This case report sheds light on a rarely described disease and provides a comprehensive approach to diagnosis and management. Primary SCCB is an extremely rare, aggressive form of breast cancer that is molecularly and histologically similar to SCLC. However, a review of the literature highlights recent mutational analyses that show important differences between these two cancer types, including an increase in PIK3CA mutations in primary SCCB. Further studies, including genomic analyses are needed to better define this malignancy and to develop a standard treatment.

## Introduction

Primary poorly differentiated neuroendocrine carcinoma/small cell carcinoma of the breast (SCCB) is a distinct subtype of breast cancer, accounting for <1% of cases of primary breast cancer. SCCB belongs to the class of tumors known as extra-pulmonary small cell carcinomas, which have also been described in the urinary bladder, prostate, esophagus, stomach, colon, rectum, gallbladder, larynx, salivary glands, cervix, and skin. The first primary case was described by Wade et al. in 1983 (1). Although it typically presents in women, there have been a few cases of primary SCCB in males reported in the literature (2). SCCB has been diagnosed in patients aged 29–81 years (3, 4) who present with a palpable breast mass. The histologic characteristics of mammary SCC are similar to SCC arising in other organs. The diagnosis of primary mammary SCC can be made if a non-mammary metastasis to the breast is excluded, or an *in situ* component is identified histologically.

Below we describe a case of a 50-year old postmenopausal women with early stage primary SCCB of the right breast without lymph node involvement. Following treatment, she remained disease-free for 3 years, but developed a right lower lobe nodule and enlarged sub-carinal lymph node, which was treated like limited small cell lung cancer (SCLC). This case is unique in that the invasive SCCB was associated with an *in situ* component which was also purely of neuroendocrine differentiation.

## Case Presentation

A 50-year-old G4P3 premenopausal South Asian woman with a history of gastroesophageal reflux disease and polycystic ovarian syndrome initially presented with a painful, palpable mass in the upper outer quadrant of the right breast noticed on self-breast exam. The patient was a lifelong non-smoker and did not report use of oral contraceptives or hormone replacement therapy. She had no personal or family history of breast or ovarian cancers and had a normal screening mammogram 1 year prior to presentation.

**Figure 1:**
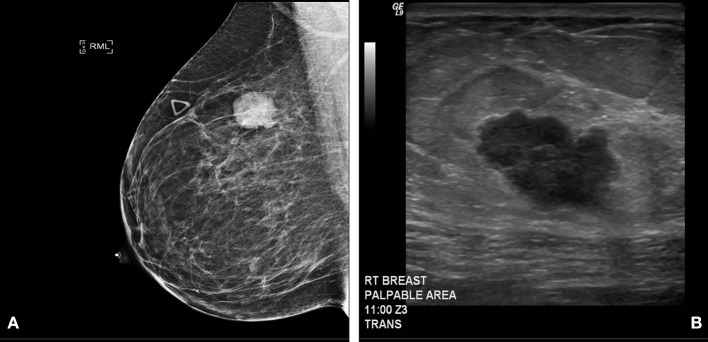
**(A)** Diagnostic Mammogram: Right medio-lateral oblique view showing an irregular high density mass in the superior central aspect of the right breast. **(B)** Ultrasonography of right breast and axilla: Irregular hypoechoic macro-lobulated mass in the superior central aspect of the right breast.

**Figure 2:**
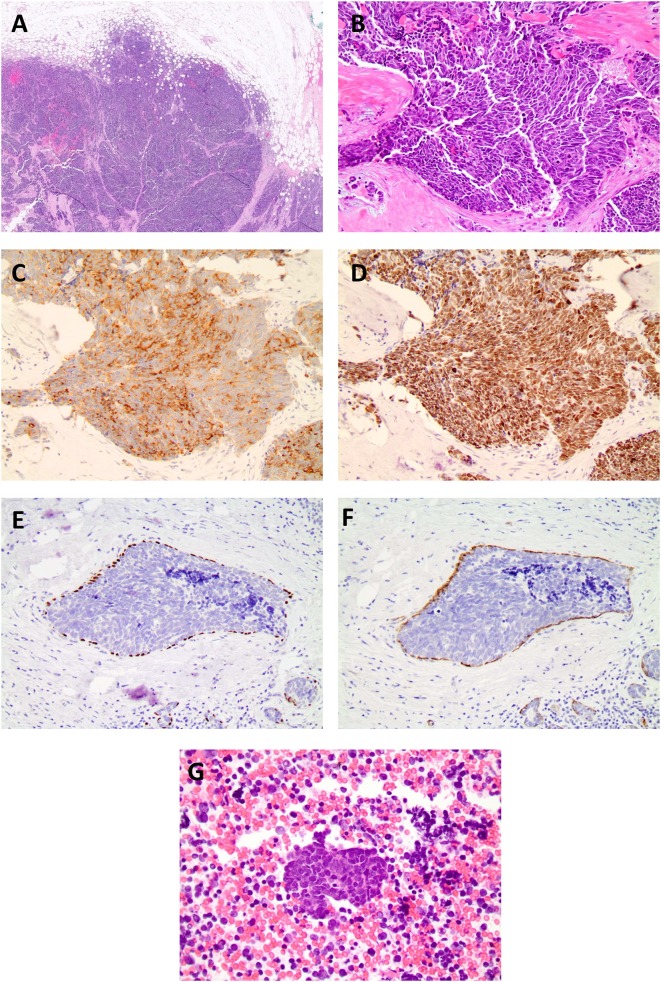
**(A)** Histopathological examination of the specimen stained on low (4x), **(B)** high power field (20x, 40x) showed neoplastic cells with high nuclear: cytoplasmic ratio, hyperchromatic nuclei, minimal cytoplasm, and indistinct nuclei, nuclear molding, high mitotic rate, consistent with the diagnosis of small cell carcinoma. Biopsy of breast cancer primary. Immuno-histochemical staining for TTF-1 synpatophysin, chromogranin, and p40 and smooth muscle myosin staining (SMMS) consistent with a diagnosis of primary SCCB, **(C)** small cell synaptophysin 20X. **(D)** small cell TTF-1 20X. **(E)** *In situ* component with neuroendocrine differentiation p40. **(F)** *in situ* component smooth muscle myosin stain. **(G)** Histopathological examination of the fine needle aspiration of the sub-carinal node revealed metastatic small cell carcinoma (Lymph node 40x).

Clinical examination revealed an irregular, firm 2.4 cm mass in the upper outer quadrant of the right breast, approximately 9.5 cm from the nipple. There were no changes of the overlying skin or nipple or palpable axillary adenopathy. Her lungs were clear to auscultation; abdominal exam was without masses and no inguinal, cervical or contralateral axillary adenopathy were palpated. Diagnostic mammogram and ultrasound (Figures 1A,B) indicated a 2.3 × 1.3 × 2.3 cm irregular mass in the upper outer quadrant of the right breast, highly suggestive of malignancy. Ultrasound guided core needle biopsy revealed poorly differentiated neuroendocrine carcinoma/small cell carcinoma (Figures 2A,B), estrogen receptor (ER) negative, progesterone receptor (PR) positive (10% weak intensity), and human epidermal growth receptor 2 (HER2) negative. Immuno-histochemical staining was positive for thyroid transcription factor 1 (TTF-1), synaptophysin and chromogranin A, and rare cells positive for GATA3, consistent with primary SCCB (Figures 2C,D). Importantly, and uniquely, an *in situ* component was identified (highlighted by the myoepithelial marker p40) which was also of purely neuroendocrine differentiation, which was conclusive evidence that this was a primary SCC of the breast. Due to the rarity of the case, these findings were confirmed at MD Anderson Cancer Center.

**Figure 3:**
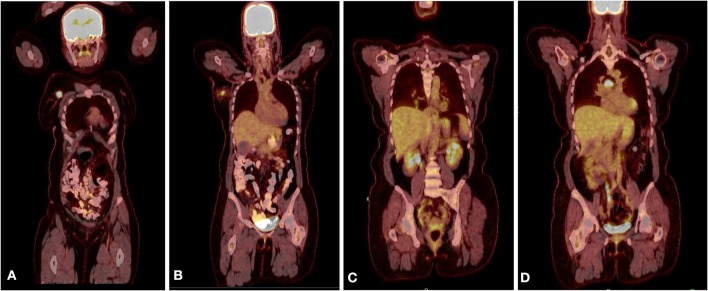
**(A)** PET CT scan showing the primary lesion in the right upper outer quadrant of the right breast. **(B)** A hyper metabolic right axillary lymph node, which is compatible with the patient's known right breast primary malignancy and a presumed metastatic right axillary lymph node. **(C)** PET scan after recurrence showing new lung nodules with FDG uptake. **(D)** PET scan after recurrence showing sub-carinal lymph node with new intense focus of FDG uptake.

Radiologic workup was conducted to exclude a non-mammary primary tumor origin and to complete staging for the breast cancer. Additionally, a positron emission tomography (PET) computerized tomography (CT) scans showed no evidence of lung, pancreatic, adrenal, or pelvic masses (Figures 3A,B). Plasma neuropeptide levels revealed elevations in gastrin (207 pg/ml; normal 0–125 pg/ml), substance P (382 pg/ml; normal 0–240 pg/ml) and neurotensin (306 pg/ml; normal <100 pg/ml). Plasma glucagon levels were also elevated (plasma neuropeptides or tumor markers are not required for the diagnosis). Germline genetic testing revealed a MUTYH E480 non-sense mutation which is associated with autosomal recessive MUTYH associated polyposis and would not be pathogenic when heterozygous with wild-type.

Based on the early clinical stage, the patient underwent a right lumpectomy with axillary lymph node dissection. Pathology revealed a poorly differentiated neuroendocrine carcinoma/ small cell carcinoma, ER/PR and HER2, were repeated on the resection specimen and were all negative with Ki-67 proliferation index of 70%. In addition, a high grade neuroendocrine *in situ* component was identified (Figures 2E,F). Immuno-histochemical staining was positive for chromogranin A, CD56, synaptophysin and TTF-1 and, notably, the *in-situ* component was also positive for TTF-1. Tumor infiltrating lymphocytes (TILs) were low positive for PD-1 staining (1–24%) and tumor cells and TILs were negative for PD-L1 staining (antibody used for PD-L1 was clone 22C3 through Foundation One). The surgical margins were free of tumor and twelve lymph nodes were negative. The patient was diagnosed with primary SCCB (stage IIA).

Foundation One testing for mutations in thirty-five genes revealed the presence of a pathogenic variant in the MUTYH gene (c.1438G>T, p.Glu480^*^), loss of exons 19-27 in RB1, SMARCA4 P1975 mutation, and Tp53 R110_L111insL mutation as well as amplification in PIK3CA, FLT3, MYC, SOX2 and CDK8. She subsequently received adjuvant chemotherapy with four cycles of cisplatin 80 mg/m^2^ on day 1 and etoposide 100 mg/m^2^ on days 1, 2, and 3 of a 21-day cycle. She received right whole breast radiation therapy in the form of 5000cGy in 25 fractions followed by a 1000cGy boost in 5 fractions (6000cGy cumulative) to the surgical site.

Following treatment, the patient remained disease-free for 3 years, as evidenced by lack of clinical evidence of recurrence and negative imaging. Routine follow-up CT scans of the thorax showed a right lower lobe nodule and enlarged sub-carinal lymph node, which showed FDG avidity by PET scan (Figures 3C,D). Bronchoscopy and trans-bronchial fine needle aspiration of the sub-carinal node revealed metastatic small cell carcinoma with Ki-67 staining in >90% of cells (Figure 2G). PD-L1 tumor proportion score was 0%. Repeat Foundation One testing performed on the slide from the sub-carinal lymph node revealed microsatellite stable status with a tumor mutation burden of 4 mutations/Mb, with amplifications noted in the CDK8, EPHB1, FLT3, MYC, PIK3CA, PIK3CB, RAD21 and SOX2 and loss of Rb and a MUTYH p E466 non-sense mutation.

She was treated with one cycle of cisplatin (80 mg/m^2^ on day 1) and etoposide 100mg/m^2^ on day 1-3) followed by one cycle concurrent with 45Gy external beam irradiation to the right lower lobe and mediastinum in 30 twice daily fractions. Follow-up CT scan of the chest revealed resolution of the right lower lobe nodule with residual scarring and significant decrease in size of the previously enlarged sub-carinal lymph node. She was then treated with two cycles of cisplatin-etoposide and two cycles of carboplatin-etoposide and concurrent atezolizumab, similar to the regimen used in the IMpower133 trial (5) for extensive stage small cell lung cancer but without maintenance atezolizumab, with radiologically and clinically stable disease and the MRI brain had no evidence of CNS metastases.

## Discussion

Primary SCCB is a rare, aggressive form of breast carcinoma, with only 56 cases reported in the literature (Table 1). It typically presents as a palpable breast or axillary mass in women over the age of 60, with lymph node involvement in 50–67% of cases at the time of diagnosis (33). Primary SCCB has similar histologic, morphologic and immune-histochemical features to SCLC, including the expression of TTF-1 in up to 50% of cases. The diagnosis of SCCB requires the clinical exclusion of a metastasis from a non-mammary primary site, or identification of an *in situ* component.

**Table 1:** Case reports of primary small cell neuroendocrine breast carcinoma.

**References**	**Age at diagnosis**	**Stage at diagnosis**	**Primary treatment**	**Adjuvant treatment**	**Outcome**	**Follow up (months)**
Wade et al. (1)	52	4 (mets to liver)	Modified radical mastectomy	Doxorubicin, vincristine, cyclophosphomide	Death from metastatic disease	9
Jundt et al. (6)	52 (male)	4	Chemo/XRT	None	Died from metastatic disease to the spine	14
Papotti et al. (7)	50	3	Mastectomy	Streptozotocin	Died from metastatic disease	14
	68	3	Mastectomy	Tamoxifen	Died from cerebral hemorrhage	9
	41	3	Mastectomy	XRT	Died from metastatic disease	15
	64	1	Lumpectomy	None	Alive without disease	44
Francois et al. (8)	68	2	Modified radical mastectomy	Chemo, XRT	Death from metastatic disease	21
Chua et al. (9)	45	2	Lumpectomy	NR	NR	NR
Fukunaga and Ushigome (10)	56	3	Mastectomy	None	Alive without disease	48
Sebenik et al. (11)	67	2	Chemotherapy	XRT	Alive without disease	33
Samli et al. (12)	60	3	Fluorouracil, epirubicine, and cyclophosphamide	Left modified radical mastectomy, axillary lymph node dissection, XRT, cisplatin/etoposide + FEC (x 4 cycles)	Alive with metastatic disease	9
Shin et al. (13)	64	1	Lumpectomy	Chemo	Alive without disease	10
	57	2	Mastectomy	Chemo	Alive without disease	10
	44	2	Lumpectomy	Chemo/XRT	Alive without disease	27
	62	3	Neo-adjuvant chemo	Mastectomy, adjuvant chemo, tamoxifen	Alive with metastatic disease	32
	70	3	Lumpectomy	Chemo/XRT	Alive without disease	3
	46	3	Mastectomy	Chemo	Alive with metastatic disease	11
	51	1	Lumpectomy	XRT	Alive without disease	25
	43	1	Lumpectomy	XRT	alive without disease	30
	50	3	Lumpectomy	Chemo/tamoxifen	Alive without disease	35
Yamasaki et al. (14)	41	2	Mastectomy	Cyclophosphamide, methotrexate, fluorouracil	Alive without disease	16
Hoang et al. (15)	41	NR	Mastectomy	NR	NR	NR
	51	NR	Lumpectomy	NR	NR	NR
Salmo and Connolly (16)	46	2	Lumpectomy	Etoposide/cisplatin, XRT	Alive without disease	9
Bergman et al. (17)	61	3	mastectomy	NR	NR	NR
Bigotti et al. (18)	56	3	Chemotherapy	Modified radical mastectomy, chemo	Death	14
Jochems and Tjalma (19)	71	2	Mastectomy	Tamoxifen	Alive without disease	12
Mariscal et al. (20)	53	3	Cisplatin + etoposide	Lumpectomy	Alive without disease	6
Sridhar et al. (21)	58	3	Lumpectomy	Adriamycin, cisplatin, XRT	Alive without disease	18
Yamamoto et al. (22)	53	3	Mastectomy	None	Alive without disease	34
	75	3	Mastectomy	CMF, tamoxifen	Alive without disease	43
Adegbola et al. (23)	46	1	Lumpectomy	XRT, cisplatin+etoposide	Alive without disease	48
	60	1	Lumpectomy	XRT, cisplatin+etoposide	Died from disease recurrence	26
	61	3	Lumpectomy	XRT, cisplatin+etoposide	Alive with metastatic disease	6
Cabibi et al. (24)	40	2	Lumpectomy	NR	NR	NR
Stein et al. (25)	54	3	Cisplatin+etoposide	Lumpectomy, XRT	Alive without disease	24
Kitakata et al. (26)	44	3	Modified radical mastectomy	Epirubicin/cyclophosphomide	Alive without disease	22
Shaco-Levy et al. (27)	28	1	Lumpectomy	Chemo, XRT	NR	NR
Kinoshita et al. (28)	31	3	Adriamycin+docetaxel	Modified radical mastectomy	Died from metastatic disease	6
Sadanaga et al. (29)	33	2	Mastectomy	epirubicin+cyclophosphomide, XRT	Alive without disease	60
Hojo et al. (30)	60	2	Modified radical mastectomy	None	Developed recurrence with metastatic disease and died despite chemotherapy	26
Quiros Rivero et al. (31)	41	2	FAC+taxol+etoposide	Lumpectomy, XRT	Alive without disease	20
Rineer et al. (4)	81	3	Irinotecan+carboplatin	XRT	Alive with disease	26
Yamaguchi et al. (32)	51	2	Mastectomy	Paclitaxel	Alive with metastatic disease	12
Latif et al. (33)	53	2	Carboplatin+etoposide	MRM, XRT	Alive without disease	NR
Nicoletti et al. (34)	40	2	Mastectomy	Adriamycin+cytoxan, carboplatin+etoposide, tamoxifen, anastrozole	Alive without disease	96
Kawanishi et al. (35)	67	1	Lumpectomy	Anastrozole	Alive without disease	12
Boyd and Hayes (36)	50	2	Mastectomy	Docetaxel+cyclophosphamide	Alive with metastatic disease	36
Ge et al. (37)	39	2	Modified radical mastectomy	Docetaxel+carboplatin	Alive without disease	NR
Ochoa et al. (38)	25	4	Cisplatin+etoposide	Radiation to the breast	Death from metastatic disease	6
Puscas et al. (39)	50	3	Farmorubicin+ cyclophosphomide+ taxotere	Mastectomy, XRT	Alive with metastatic disease	NR
Jiang et al. (2)	79 (male)	2	Modified radical mastectomy	Irinotecan+carboplatin	Died from metastatic disease	27
Dalle et al. (40)	47	2	Mastectomy	Cisplatin+etoposide, fluorouracil+epirubicin+ cyclophosphamide, tamoxifen	Alive without disease	10
Raber et al. (41)	38	3	Carboplatin+etoposide	Modified radical mastectomy	Alive without disease	15
Tremelling et al. (42)	65	3	Carboplatin+etoposide	XRT	Alive without disease	3
	61	3	Lumpectomy	XRT, cisplatin+etoposide	Alive with metastatic disease	6

*NR, not reported*.

Due to the rarity and limited reports of primary SCCB, a standard approach to treatment is largely undefined. Treatment regimens in the literature include combinations of surgery, chemotherapy, radiation therapy, and endocrine therapy depending on tumor size and lymph node status. Hormonal therapy is added if the tumor expresses the appropriate receptors (13, 23, 43). More recent reports have described treatment with breast conservation therapy combined with either neo-adjuvant or adjuvant chemotherapy depending on the clinical scenario. Most adjuvant chemotherapy regimens include a platinum agent and etoposide, given that biologic markers of SCCB are similar to that of SCLC (see Appendix for discussion on characteristics of SCCB vs. SCLC and genomic studies in SCCB) (23, 37, 38, 44–46). However, some have reported using anthracycline and taxane based combinations typically used for invasive breast cancer (23, 33, 47, 48).

Our patient had a very high Ki-67, and she was treated with platinum based chemotherapy. The present report details a case of primary SCCB, as established with neuroendocrine markers on immuno-histochemical staining in combination with a solitary breast mass, lack of regional lymph node involvement and lack of evidence of any other masses in other organs on radiologic imaging, and importantly an *in situ* component which was of pure neuroendocrine differentiation, which is a rare finding. The patient was treated with breast conservation surgery, followed by systemic chemotherapy using carboplatin and etoposide along with radiation therapy. Unfortunately, the patient had a recurrence after 3 years with genomic studies indicating shared mutations consistent with a single primary and was treated with chemo-radiation similar to limited stage SCLC and is now in clinical remission. Her tumor also expressed an amplification of PIK3CA, which may serve as a therapeutic target in the future.

This case report sheds light on a rarely described disease and provides a comprehensive approach to diagnosis and management. Further studies, including genomic analyses are needed to better define this malignancy and to develop a standard treatment.

## Data Availability Statement

All datasets generated for this study are included in the article/Supplementary Material.

## Ethics Statement

The studies involving human participants were reviewed and approved by Institutional Review Board, The Ohio State University, Wexner Medical Center. The patients/participants provided their written informed consent to participate in this study. Written informed consent was obtained from the individual(s) for the publication of any potentially identifiable images or data included in this article.

## Author Contributions

HB, MK, EM, SF, and MC prepared the body of the manuscript. BR, GT, JW, MS, JV, SS, NW, DS, ML, RW, VP, TW, BK, and DC critically reviewed the publication. All authors endorsed the final form of the manuscript.

## Conflict of Interest

The authors declare that the research was conducted in the absence of any commercial or financial relationships that could be construed as a potential conflict of interest.

## Abbreviations

CT: Computerized Tomography
ER: Estrogen Receptor
HER2: Human Epidermal Growth Factor Receptor 2
PET: Positron Emission Tomography
PR: Progesterone Receptor
SCCB: Small cell Carcinoma of the Breast
SCLC: Small Cell Lung Cancer
TTF1: Thyroid Transcription Factor 1.
