# Supplementary material for: TTF-1 Positive Primary Small Cell Carcinoma of the Breast: A Case Report and Review of the Literature
Source: Front Endocrinol (Lausanne). 2020 Apr 29;11:228. doi: 10.3389/fendo.2020.00228 (PMC7201766; doi:10.3389/fendo.2020.00228)
Supplement: Supplementary file 1 [file Data_Sheet_1.docx]

**Appendix**

Primary SCCB has similar histologic, morphologic and immune-histochemical features to small cell lung carcinoma (SCLC), including the expression of TTF-1 in up-to 50% of cases, making it difficult to differentiate at diagnosis. As a result, thorough evaluation with CT or PET imaging is necessary to confirm the absence of an alternative primary site. Immuno-histochemical staining for neuroendocrine markers, specifically chromogranin A and synaptophysin, is often positive, is but is not necessary to make the diagnosis ^1,2^. Electron microscopy can identify neurosecretory granules, which are characteristic of SCCB, although it is not required for the diagnosis. Other diagnostic features that support a primary breast origin include the presence of associated ductal carcinoma in situ (DCIS) and presence of regional lymph node metastases ^3^. Estrogen receptors are positive in 35-54% of primary SCNCB, while HER2/neu is almost always negative ^4,5^.

Based on reviews of prior cases, it seems that while SCCB shares histologic and morphologic features with small cell lung cancer (SCLC), it bears more of a clinical resemblance to more common forms of breast cancer ^1,6,7^. Some studies show that primary SCCB has a more favorable prognosis than SCLC if caught in the early stages of disease while still localized ^1,7-15^. Two studies, a population-based study from the UK and a study using the SEER registry, both showed that SCCB has the highest overall five and ten year survival outcomes among all cases of extra-pulmonary small cell carcinomas (EPSCC) ^16,17^. This improved survival may be due to the fact that SCCB often presents at earlier stages as compared to patients with SCLC translating into an overall better prognosis ^17^. This highlights the importance of timely detection because once the cancer metastasizes, the median overall survival drastically declines and is no longer appreciably different from that of SCLC.

A recent study by McCullar, et al. used immunohistochemistry staining, in situ hybridization, next generation sequencing and Sanger sequencing to further characterize the differences between primary SCCB and SCLC. Primary SCCB was found to be more commonly hormone receptor-positive than SCLC, and next generation sequencing demonstrated that 33% of the patients with primary SCCB expressed PIK3CA mutations in contrast to 0% of SCLC patients. There was similarity in TP53 mutations with 75% of both expressing mutations and similarity in immunohistochemistry for PDL1 (0 v 0%) and PD1 (50 v 42%) ^18^. This study also addressed the use of drugs targeting the PI3K/AKT pathway, including inhibitors of rapamycin, as potential therapeutic agents for the treatment of primary SCCB.

Although large scale genomic studies of cancer have been key to the development of new therapies, there has been very little focus on the genomic landscape of primary breast neuroendocrine carcinomas, given the limited number of cases. While most case reports have focused on immune-histochemical staining for genetic analysis, two have reported the use of targeted sequencing panels in patients with neuroendocrine breast cancer and identified recurrent mutations affecting PIK3CA, the FGFR family and chromatin remodeling genes ^19,20^. In another study, the majority of primary SCCB were found to express TOP2A, highlighting a potential role for TOP2A-targeted therapy, including topoisomerase 2 inhibitors such as etoposide and anthracyclines. Additionally, there has been a case reported of a patient with primary SCCB who achieved a good clinical response with anthracycline therapy combined with doxorubicin and cyclophosphamide followed by carboplatin and etoposide ^21^.

Overall, primary SCCB is an extremely rare, aggressive form of breast carcinoma that is molecularly and histologically similar to SCLC. However, a review of the literature highlights recent mutational analyses that show important differences between these two cancer types, including an increase in PIK3CA mutations in primary SCCB.

**References**

1. Adegbola T, Connolly C, Mortimer G: Small cell neuroendocrine carcinoma of the breast: a report of three cases and review of the literature. Journal of clinical pathology 58:775-778, 2005

2. Inno A, Bogina G, Turazza M, et al: Neuroendocrine carcinoma of the breast: current evidence and future perspectives. The oncologist 21:28-32, 2016

3. Jablon LK, Somers RG, Kim PY: Carcinoid tumor of the breast: treatment with breast conservation in three patients. Annals of surgical oncology 5:261-264, 1998

4. Latif N, Rosa M, Samian L, et al: An unusual case of primary small cell neuroendocrine carcinoma of the breast. The breast journal 16:647-651, 2010

5. Wang J, Wei B, Albarracin CT, et al: Invasive neuroendocrine carcinoma of the breast: a population-based study from the surveillance, epidemiology and end results (SEER) database. BMC cancer 14:147, 2014

6. Ge Q-D, Lv N, Cao Y, et al: A case report of primary small cell carcinoma of the breast and review of the literature. Chinese journal of cancer 31:354, 2012

7. Jochems L, Tjalma WA: Primary small cell neuroendocrine tumour of the breast. European Journal of Obstetrics & Gynecology and Reproductive Biology 115:231-233, 2004

8. Adams R, Dyson P, Barthelmes L: Neuroendocrine breast tumours: breast cancer or neuroendocrine cancer presenting in the breast? The Breast 23:120-127, 2014

9. Shin SJ, DeLellis RA, Ying L, et al: Small cell carcinoma of the breast: a clinicopathologic and immunohistochemical study of nine patients. The American journal of surgical pathology 24:1231-1238, 2000

10. Rovera F, Masciocchi P, Coglitore A, et al: Neuroendocrine carcinomas of the breast. International Journal of Surgery 6:S113-S115, 2008

11. Yerushalmi R, Hayes M, Gelmon K: Breast carcinoma—rare types: review of the literature. Annals of oncology 20:1763-1770, 2009

12. Dalle IA, Abbas J, Boulos F, et al: Primary small cell carcinoma of the breast: a case report. Journal of medical case reports 11:290, 2017

13. Sata N, Tsukahara M, Koizumi M, et al: Primary small-cell neuroendocrine carcinoma of the duodenum–a case report and review of literature. World journal of surgical oncology 2:28, 2004

14. Raber B, Dao T, Howard E, et al: Primary small-cell carcinoma of the breast, Baylor University Medical Center Proceedings, Taylor & Francis, 2017, pp 200-202

15. Tachibana K, Abe N, Abe S, et al: A Case of Primary Poorly Differentiated/Small Cell Carcinoma of the Breast in a Patient with Von Recklinghausen's Disease. Gan to kagaku ryoho. Cancer & chemotherapy 43:2022-2025, 2016

16. Grossman RA, Pedroso FE, Byrne MM, et al: Does surgery or radiation therapy impact survival for patients with extrapulmonary small cell cancers? Journal of surgical oncology 104:604-612, 2011

17. Wong YNS, Jack RH, Mak V, et al: The epidemiology and survival of extrapulmonary small cell carcinoma in South East England, 1970–2004. BMC cancer 9:209, 2009

18. McCullar B, Pandey M, Yaghmour G, et al: Genomic landscape of small cell carcinoma of the breast contrasted to small cell carcinoma of the lung. Breast cancer research and treatment 158:195-202, 2016

19. Marchiò C, Geyer FC, Ng CK, et al: The genetic landscape of breast carcinomas with neuroendocrine differentiation. The Journal of pathology 241:405-419, 2017

20. Ang D, Ballard M, Beadling C, et al: Novel mutations in neuroendocrine carcinoma of the breast: possible therapeutic targets. Applied Immunohistochemistry & Molecular Morphology 23:97-103, 2015

21. Encinas G, Maistro S, Pasini FS, et al: Somatic mutations in breast and serous ovarian cancer young patients: a systematic review and meta-analysis. Revista da Associação Médica Brasileira 61:474-483, 2015
